# Synthesis of Gold Functionalised Nanoparticles with the *Eranthis hyemalis* Lectin and Preliminary Toxicological Studies on *Caenorhabditis elegans*

**DOI:** 10.3390/ma11081363

**Published:** 2018-08-06

**Authors:** Jamila Djafari, Marie T. McConnell, Hugo M. Santos, José Luis Capelo, Emilia Bertolo, Simon C. Harvey, Carlos Lodeiro, Javier Fernández-Lodeiro

**Affiliations:** 1 BIOSCOPE Group, LAQV@REQUIMTE, Chemistry Department, Faculty of Science and Technology, University NOVA of Lisbon, Caparica Campus, 2829-516 Caparica, Portugal; j.djafari@fct.unl.pt (J.D.); hms14862@fct.unl.pt (H.M.S.); jlcm@fct.unl.pt (J.L.C.); cle@fct.unl.pt (C.L.); 2 PROTEOMASS Scientific Society, Rua dos Inventores, Madam Parque, Caparica Campus, 2829-516 Caparica, Portugal; 3 Biomolecular Research Group, School of Human and Life Sciences, Canterbury Christ Church University, Canterbury CT1 1QU, UK; mm465@outlook.com (M.T.M.); simon.harvey@canterbury.ac.uk (S.C.H.)

**Keywords:** *Caenorhabditis elegans*, toxicity, gold nanoparticles, nanocomposites, lectin protein, Reactive Oxygen Species (ROS)

## Abstract

The lectin found in the tubers of the Winter Aconite (*Eranthis hyemalis*) plant (EHL) is a Type II Ribosome Inactivating Protein (RIP). Type II RIPs have shown anti-cancer properties and have great potential as therapeutic agents. Similarly, colloidal gold nanoparticles are successfully used in biomedical applications as they can be functionalised with ligands with high affinity and specificity for target cells to create therapeutic and imaging agents. Here we present the synthesis and characterization of gold nanoparticles conjugated with EHL and the results of a set of initial assays to establish whether the biological effect of EHL is altered by the conjugation. Gold nanoparticles functionalised with EHL (AuNPs@EHL) were successfully synthesised by bioconjugation with citrate gold nanoparticles (AuNPs@Citrate). The conjugates were analysed by UV-Vis spectroscopy, Dynamic Light Scattering (DLS), Zeta Potential analysis, and Transmission Electron Microscopy (TEM). Results indicate that an optimal functionalisation was achieved with the addition of 100 µL of EHL (concentration 1090 ± 40 µg/mL) over 5 mL of AuNPs (concentration [Au^0^] = 0.8 mM). Biological assays on the effect of AuNPs@EHL were undertaken on *Caenorhabditis elegans*, a free-living nematode commonly used for toxicological studies, that has previously been shown to be strongly affected by EHL. Citrate gold nanoparticles did not have any obvious effect on the nematodes. For first larval stage (L1) nematodes, AuNPs@EHL showed a lower biological effect than EHL. For L4 stage, pre-adult nematodes, both EHL alone and AuNPs@EHL delayed the onset of reproduction and reduced fecundity. These assays indicate that EHL can be conjugated to gold nanoparticles and retain elements of biocidal activity.

## 1. Introduction

Lectins are a class of proteins ubiquitously expressed in plants, animals, bacteria, and viruses. They are well known for their ability to agglutinate erythrocytes, and their ability to bind carbohydrates selectively based on the individual sugar specificity of the lectin [1,2]. Plant lectins play a key role in plants’ defences [3,4]; insecticidal, antifungal, and antiviral qualities have also been widely described [5,6,7,8]. *Eranthis hyemalis* (Winter Aconite) is a late winter/early spring flowering perennial plant of the family Ranunculaceae. *E. hyemalis* possesses a proteinaceous toxin (named *Eranthis hyemalis* lectin, EHL), found to cause agglutination of erythrocytes as well as impacting on the fitness of some agricultural pests and plant viruses [5,9]. To date, *E. hyemalis* is the sole representative of the Ranunculaceae to be reported to express lectin activity. Due to the structural and toxicity studies conducted [5,9,10,11], EHL should be classed as a Type II Ribosome Inactivating Protein (RIP). RIPs are a class of enzymes (EC 3.2.2.22) with a mode of action which results in the breakage of a glycosidic bond in the 28s rRNA in the 60S subunit of the ribosome, resulting in the disruption of protein synthesis and subsequent cell death. EHL shows specificity for *N*-acetyl-galactosamine [5,9], an overexpressed and incompletely glycosylated sugar in the Tn antigen which characterizes cancer linked *O*-glycans [12]. Other *N*-acetyl-galactosamine specific RIPs such as the Mistletoe lectin and Riproximin have demonstrated promising therapeutic relevance as anticancer agents [13,14,15]. EHL could therefore have a promising future as an anticancer agent, if its toxicity can be harnessed and tuned to appropriate levels.

Previous work by some of the authors has shown biocidal effects of EHL against the free-living nematode *Caenorhabditis elegans* [16]. *C. elegans* is a well-established model organism for initial toxicological studies due to the conserved nature of its biological and biochemical processes, including the stress response and disease pathways [17]. A wide range of available mutants, a short life cycle, a well-documented life history, and a largely transparent body (which makes it possible to observe unusual effects easily), are some of the advantages of *C. elegans* [18]. Developing *C. elegans* individuals pass through a well-defined set of life stages, with individuals hatching as first larval stage (L1) worms. These L1 worms subsequently molt through three further larval stages—the L2, L3, and L4 stages—before maturing as adults. Development is temperature dependent and takes approximately 3 days at 20 °C. Newly hatched L1 worms measure around 0.25 mm in length, and in their adult stage they reach up to 1 mm. An interesting characteristic of *C. elegans* is its ability to enter an alternate L3 life cycle stage known as the dauer larvae. Naturally induced dauer larval arrest occurs when L1 and L2 larva are exposed to environments not suited for growth and reproduction [19]. These environments are characterised by a depleted food source and population overcrowding, with the chemosensory cues and signals for these detected by the L1 larvae. As part of development into dauer larvae, worms develop a specialized outer cuticle, and seal their mouths, preventing feeding. In combination with changes in their metabolism, these adaptations mean that dauer larvae have an increased lifespan, an enhanced resistance to environmental stress and are resistant to many chemical treatments that would kill other lifecycle stages. This resulting dauer larvae is in an arrested developmental state (a temporary halt in its development), and can survive for months until conditions improve, at which point development resumes with dauer larvae moulting in to L4s [19].

Colloidal gold nanoparticles (AuNPs) have long been exploited in science for their optical properties. The applications of AuNPs have increased enormously in recent years and are used routinely in both material science and within biomedical sciences as bioimaging agents, therapeutic agents, and drug delivery vehicles [20,21,22,23]. AuNPs can be functionalized with both therapeutic and imaging agents simultaneously, thus are a powerful tool in cellular studies [24]. Gold nanoparticles can be functionalized with ligands with high affinity and specificity for target cells such as the Tn antigen, which is where conjugating gold nanoparticles with a RIP such as EHL presents an opportunity to fine tune EHL’s biological effects. 

Herein we present the synthesis and characterization of AuNPs conjugated with EHL (AuNPs@EHL), and the preliminary study of the effects of AuNPs@EHL on *C. elegans*. The aim was to establish the viability of the conjugate and to perform a set of initial assays to establish if the biological effect of EHL is altered by the conjugation. The conjugates were analysed by UV-Vis spectroscopy, Dynamic Light Scattering (DLS), Zeta Potential analysis, and Transmission Electron Microscopy (TEM). Biological assays on the effect of AuNPs@EHL on *C. elegans* were performed using first life stage (L1) and pre-adult stage (L4) nematodes, and the results compared to previously published data on the effect of EHL on L1 nematodes [16]. Additionally, the effects of naked AuNPs on L1 and L4 nematodes, and the effect of EHL on L4s were also studied.

## 2. Materials and Methods

### 2.1. Materials

Tetrachloroauric (III) acid (HAuCl_4_·3H_2_O), Sodium hydroxide (NaOH), Hydrochloric acid (HCl), Sodium chloride (NaCl), and Sodium citrate tribasic (C_6_H_5_Na_3_O_7_·2H_2_O) were purchased from Sigma Aldrich (St. Louis, MO, USA), Strem Chemicals (Newton, UK), Fluka (St. Louis, MO, USA) or Panreac (Barcelona, Spain), and used without further purifications. Acetonitrile (CAN, 99.9% purity) and acetic acid glacial (99.7% purity) were purchased from Sigma-Aldrich (St. Louis, MO, USA). Ethanol (EtOH, 96% purity) was purchased in Panreac (Barcelona, Spain). Water was always Milli-Q grade by Millipore.

Protein quantification was accomplished by measuring the absorbance at 595 nm with the use of a Bradford Protein Assay using a CLARIOStar^®^ High performance monochromator multimode BMG Labtech, Germany from the Proteomass-BIOSCOPE Facility lab. The transmission electron microscopy (TEM) images were obtained using a JEOL JEM 1010F transmission electron microscope from the CACTI, University of Vigo, (Spain), operating at 100 kV. Samples were prepared dropping 5 μL of the colloidal suspension on a copper grid coated with a continuous carbon film, and the solvent was allowed to evaporate. TEM Images were characterized using ImageJ software (Image 1.51 h, Wayne Rasband, National Institutes of Health, Bethesda, MD, USA) [25], with a minimum of three hundred nanoparticles measured. The size measurements were performed with the nanoparticles diluted in 1 mL of water in a Zetasizer Nano ZS instrument (Malvern Instruments, Malvern, Panalytical, UK) in the PROTEOMASS facilities. Zeta potential quantification was carried out in the same Zetasizer Nano ZS instrument. 

### 2.2. Synthesis of EHL Conjugated Gold Nanoparticles (AuNPs@EHL)

The AuNPs@Citrate were prepared by modification of a previously published protocol [26]. An aqueous solution (125 mL) containing 49 mg of tetrachloroauric (III) acid (HAuCl_4_·3H_2_O) was heated until boiling point without reflux to ensure a low temperature gradient in the walls of the flask; the solution was kept boiling for 10 min. Then, a pre-boiled aqueous solution (12.5 mL) containing 147 mg of sodium citrate tribasic (C_6_H_5_Na_3_O_7_·2H_2_O) was added rapidly. The reaction mixture was heated to boiling for an additional 15 min, and then allowed to cool to ~25 °C and left with magnetic stirring overnight. The reaction was then diluted to a final volume of 140 mL with milliQ water and was transferred into a glass bottle for storage. The final obtained citrate gold nanoparticles (AuNPs@Citrate) presented a concentration of 0.8 mM in terms of Au (0) (See SI for details, Figure S1) and were used without purification.

To achieve the bioconjugation of the AuNPs@Citrate with EHL, the protein was suspended in Phosphate Buffer Saline (PBS) solution. Quantification via Bradford technique indicated an EHL concentration of 1090 ± 40 µg/mL. Six experiments were performed in order to characterize the optimal quantity of EHL to achieve the stabilization of the nanoparticles: 25 µL, 50 µL, 100 µL, 200 µL, 300 µL, and 500 µL of EHL solution were used. On each case, the EHL solution was added onto 5 mL of AuNPs@Citrate ([Au (0)] = 0.8 mM) and left under vigorous stirring at room temperature for 2 h, to ensure effective functionalization. The nanoparticles obtained—AuNPs@EHL-1 (25 µL), AuNPs@EHL-2 (50 µL), AuNPs@EHL-3 (100 µL), AuNPs@EHL-4 (200 µL), AuNPs@EHL-5 (300 µL), AuNPs@EHL-6 (500 µL)—were isolated by centrifugation at 14,000 rpm during 25 min, and then suspended in PBS solution. A second centrifugation cycle and resuspension in 5 mL of MilliQ water were performed. The first supernatant was filtered in a cellulose filter of 0.22 µm, and quantified by the Bradford technique, in order to determine EHL concentration at the nanoparticles surface. AuNPs@EHL-3 (100 µL) was selected to perform the biological studies (See Figures S2 and S3 in the Supplementary Information, SI).

### 2.3. Nematode Assay

Worms were obtained from the *Caenorhabditis* Genetics Center and maintained using standard methods [27] on nematode growth media plates (NGM) using an *Escherichia coli* OP50 strain food source. *C. elegans* strain N2 was used for the assays. In all experiments, treatments and genotypes were blind coded, the position of plates within experimental blocks was randomised, and any contaminated plates displaying evidence of fungal growth were excluded from all analysis. Assays were initiated using arrested and synchronised *C. elegans* first stage larvae (L1s) obtained by allowing eggs, isolated from gravid hermaphrodites by hypochlorite treatment [27], to hatch on NGM plates in the absence of food for 24 h at 20 °C.

For the experiments on L1 stage worms, synchronised L1s were incubated in 15 mL eppendorf tubes at 20 °C in a solution of one of four treatments (see below) for 6 h. Three replicates of each treatment were made. After incubation, all treatments were subjected to a cycle of three washes with M9 buffer [27] with a 2 min centrifugal spin at 2000 *g*. Worms were then added to seeded (*Escherichia coli* OP50) NGM plates and incubated at 20 °C. Plates were then scored on day 3 for survival, arrested development, and for dauer larvae formation.

For the experiments on L4 stage worms, synchronised L1s were placed on seeded NGM plates and incubated at 20 °C until the L4 stage was reached. Treatment was then carried out in the same set of liquid conditions as experiment 1 for 18 h, with tubes placed in a shaking incubator at 20 °C overnight. As previously, worms were then washed three times and moved onto *en masse* onto NGM plates (one plate per tube). L4 stage worms were then individually picked from these plates onto seeded NGM plates, 50 per treatment. Worms were then transferred to new plates daily during the reproductive period, with progeny allowed to develop for 2 days before they were counted. Treatments were as follows: First, M9 liquid nutrient media and EHL @ [1.51 mg/mL]; second, M9 liquid nutrient media and AuNPs@EHL-3; third, M9 liquid nutrient media and AuNPs@EHL; and fourth, M9 liquid nutrient media.

## 3. Results and Discussion

### 3.1. Synthesis and Characterization of the Bioconjugated Gold Nanoparticles (AuNPs@EHL)

**Figure 1:**
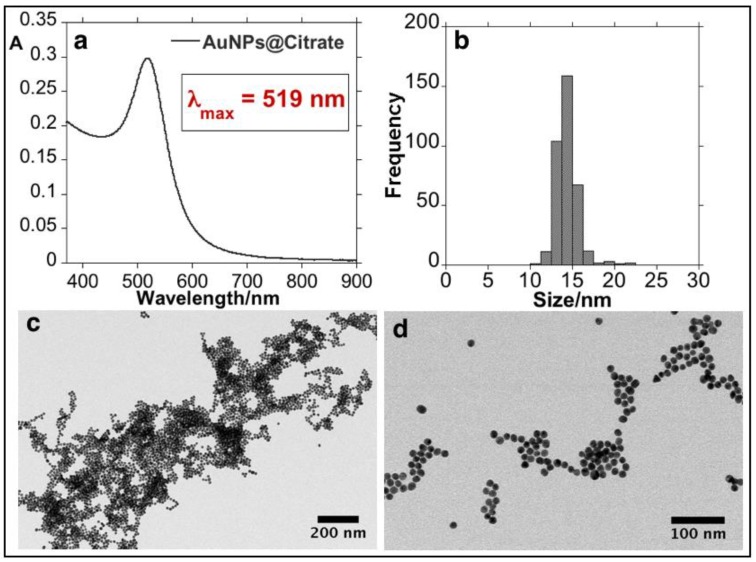
UV-Vis spectrum (**a**) histogram (**b**) and low magnification TEM images (**c**,**d**) of AuNPs@Citrate. The histogram is derived from measurements of 300 nanoparticles made in ImageJ software.

AuNPs@EHL were synthesized by attaching the protein to the nanoparticles surface through adsorption. This methodology has been widely adopted to prepare many nanoparticles/protein bioconjugates [28,29,30,31]. In our case, the bioconjugation was achieved by incubating the AuNPs@Citrate in water solution, with an EHL solution in phosphate-buffered saline (PBS). The particles were analyzed by UV-Vis spectroscopy (JASCO Co., Tokyo, Japan), DLS (MALVERN, Panalytical, UK), Zeta Potential (MALVERN, Panalytical, UK), and TEM (JEM 1010, JEOL, Tokyo, Japan) analysis. The ruby red colloidal solution of AuNPs@Citrate presents a Localized Surface Plasmon Resonance (LSPR) band at 519 nm in the UV-Vis spectrum (Figure S1). TEM analysis shows that the spherical AuNPs@Citrate have an average size 14.4 nm (SD = 1.3) (see Figure 1). DLS experiments indicated that the AuNPs@Citrate measured 18.80 nm in Z-average, with a Zeta Potential equal to −43.6 mV/cm, confirming the nanoparticles stabilization by citrate molecules. The concentration of the gold colloid obtained, in terms of Au (0), was calculated from the absorption at 400 nm [32,33,34]. Thus, we obtained a concentration of [Au (0)] = 0.8 mM (see SI for details).

An incubation process was performed to conjugate the AuNPs@Citrate with EHL; the concentration of the EHL solution used (determined by the Bradford technique) was 1090 ± 40 µg/mL. Six experiments were performed in order to characterize the optimal quantity of EHL to achieve the stabilization of the nanoparticles. The following volumes of EHL solution were used: 25 µL (AuNPs@EHL-1), 50 µL (AuNPs@EHL-2), 100 µL (AuNPs@EHL-3), 200 µL (AuNPs@EHL-4), 300 µL (AuNPs@EHL-5), and 500 µL (AuNPs@EHL-6). All the nanoparticles obtained were characterized by UV-Vis spectroscopy, DLS, Zeta Potential, and TEM. Results are summarized in Table 1 and Figure S3 (see SI).

**Figure 2:**
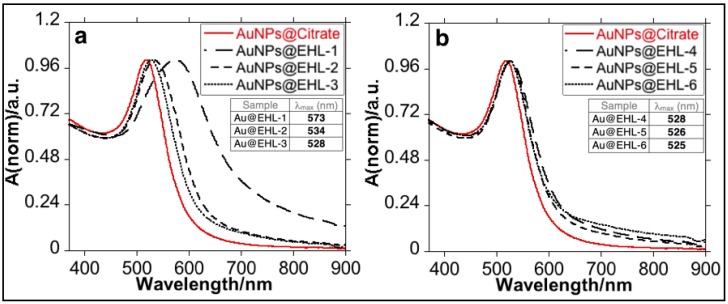
UV-Vis spectra of the different AuNPs@EHL samples synthesized: (**a**) AuNPs@EHL-1, AuNPs@EHL-2, and AuNPs@EHL-3; and (**b**) AuNPs@EHL-4, AuNPs@EHL-5, and AuNPs@EHL-6.

**Figure 3:**
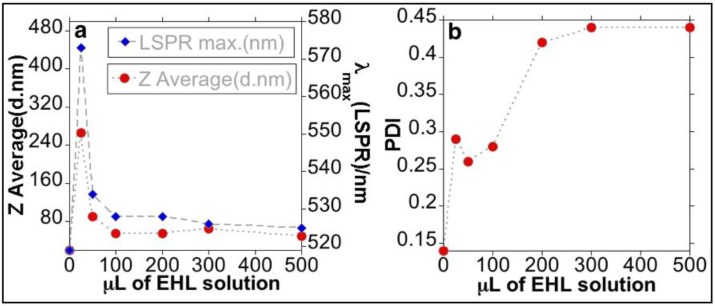
(**a**) Z-average (red dot) and LSPR (Localized Surface Plasmon Resonance) maximum (blue dot) and (**b**) PDI (polydispersity Index) of the AuNPs@EHL obtained as a function of EHL amounts added.

**Figure 4:**
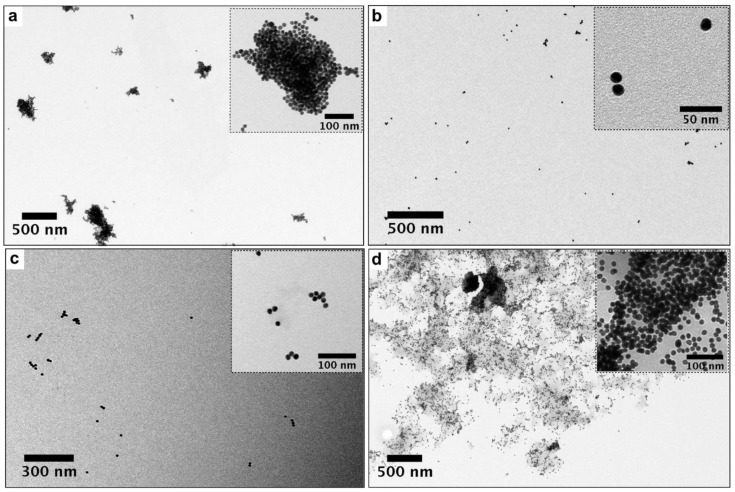
TEM images of AuNPs@EHL when different amounts of protein are added: (**a**) AuNPs@EHL-2 (25 µL), (**b**) AuNPs@EHL-3 (100 µL), (**c**) AuNPs@EHL-5 (200 µL), and (**d**) AuNPs@EHL-2 (500 µL). In all cases, the nanoparticles go through two centrifugation cycles (14,000 rpm × 25 min) and are resuspended in MilliQ water.

Addition of low quantities of EHL (AuNPs@EHL-1 and AuNPs@EHL-2) does not allow the stabilization of the colloidal system, and induces nanoparticles aggregation by partial functionalization. As it can be seen in Figure 2, a red shift on the LSPR band was observed. In addition, the aggregation of the nanoparticles was confirmed by the increase in Z-average value obtained for this samples (see Figure 3 and Figure S2 in SI). TEM images also confirm that aggregation has occurred (see Figure 4a). Due to its dimeric structure, EHL could act as a link between the nanoparticles, and thus induce aggregation in the colloidal system. Moreover, due to the incomplete formation of the nanoparticles’ corona, in the presence of PBS salts it could modify the isotropy charge produced by the citrate adsorbed in the surface, resulting in the formation of nano-aggregates.

For higher amounts of EHL (AuNPs@EHL-4, AuNPs@EHL-5, and AuNPs@EHL-6), saturation of the nanoparticles surface occurs, as shown by the similar max of the LSRP band when compared with AuNPs@EHL-3, together with the similar Z average obtained (see Figure 3 and Figure S2 in SI). Moreover, a significant increase in PDI (polydispersity index) was observed for AuNPs@EHL-4, AuNPs@EHL-5 and AuNPs@EHL-6 (see Figure 3 and Figure S2 in SI). These results suggest that the colloidal systems conjugated in these conditions were composed of AuNPs@EHL and an excess of EHL molecules. In our case, additional centrifugation cycles were not able to wash the unreacted EHL. To this respect, the decrease in the rotational speed in the time intervals studied (between 20 min and 1 h) resulted in a considerable increase in the concentration of AuNPs in the supernatant. (See Figure S3 in SI).

In an attempt to quantify the amount of protein on the surface of NPs, we have analyzed the first supernatant obtained during the purification process of the nanoparticles using the Bradford technique. For higher amounts of EHL (AuNPs@EHL-4, AuNPs@EHL-5, and AuNPs@EHL-6), this supernatant was not completely clear and still contained nanoparticles, even after filtering, which can produce an erroneous reading using the Bradford technique. This is because in spectroscopic quantification the wavelength used may overlap with the LSPR band of the AuNPs, resulting in the appearance of increased protein values (see Table 1).

The results obtained suggest that the best functionalization is achieved with the addition of 100 µL of protein (1090 ± 40 µg/mL) onto 5 mL of AuNPs@Citrate solution. The AuNPs@EHL obtained (sample AuNPs@EHL-3) show a high percentage of protein functionalized (17.7 g/mL); TEM images and DLS analysis confirm lack of aggregation and the stability of the resulting colloidal solution (See Figure 4b and Figure S2 in SI). UV-Vis spectra show a redshift in the LSPR band from 519 nm to 528 nm (see Figure 3) suggests a composition change on the surface of the nanoparticles, indicative of the protein adsorption [35]. The Z-average increased from 18.8 nm (for AuNPs@Citrate) to 54.4 nm for AuNPs@EHL-3.

**Figure 5:**
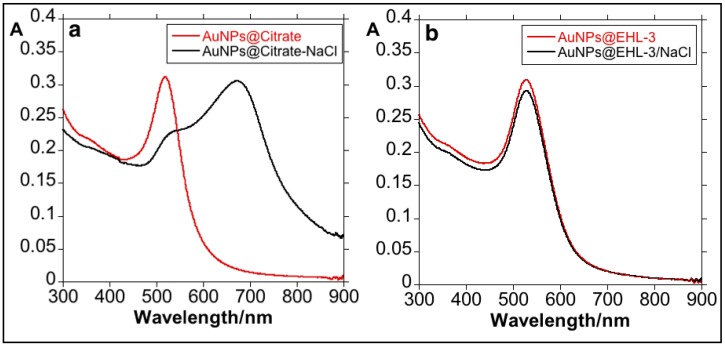
UV-Vis study of the effects of adding of 200 L NaCl 2 M to 3 mL of AuNPs@Citrate (**a**) and AuNPs@EHL-3 (**b**); dilution factor 1:10.

In order to show that the EHL is indeed conjugated to the surface of the nanoparticles, a test was carried out by adding 200 L of 2 M NaCl to a solution of 3 mL of the respective gold colloid (factor dilution 1:10); results are shown in Figure 5. When NaCl was added to the AuNPs@Citrate, the colloid aggregation occurred. This phenomenon can be visualized through the color change of the solution from red to blue accompanied by a marked red-shift in λmax of the LSRP band. Due to the presence of electrolytes such as sodium chloride, the negative charge of the colloids is masked causing an imbalance between the repulsive and attractive forces and producing colloid aggregation [36]. The addition of NaCl onto AuNPs@EHL-3 did not result in any destabilization of the system, showing that EHL molecules are on the nanoparticles surface. Analysis by DLS of the sample AuNPs@EHL-3 in 100% PBS again confirms the presence of the EHL protein on the surface of the AuNPs (see Figure S3).

### 3.2. Biological Activity against C. elegans

*C. elegans* is a well-established model in toxicological research to investigate toxicological responses at a whole organism level [37,38,39]. The nematode has also been used specifically in relation to the toxicology of nanoparticles [40,41]. The effect of AuNPs@EHL on *C. elegans* was investigated and compared to the effect of AuNPs@Citrate treatment and EHL treatment. The biological studies were performed using AuNPs@EHL-3 (which from now on will be referred to as AuNPs@EHL). Previous work by some of the authors has shown that EHL has biocidal properties against *C. elegans*, producing a significant reduction in fecundity, development and growth. Additionally, the authors reported a high incidence of abnormal dauer development when arrested L1 larvae were treated with EHL and then maintained on food, i.e., EHL treatment resulted in dauer larvae formation under conditions that produced 100% non-dauer development in non-treated worms. These EHL-induced dauer larvae were also unable to resume development when maintained on food. The authors called this a “lectin-induced effect” and suggested that the occurrence of dauer formation and a failure to recover in the presence of food indicates that EHL is binding specifically to amphid neurons [16].

Here, two sets of experiments were performed. The first set was a replication of the work on the effects of EHL on *C. elegans* [16], using AuNPs@EHL. The second set of experiments (set 2) were carried out using L4 stage (pre-adult) worms and had not previously been conducted with EHL. An observation that the AuNPs@EHL sample did not agglutinate erythrocytes, a characteristic of the native protein, was recorded prior to commencement of the experiments. This effect suggests that conjugation changes important properties in the native protein.

For the first set of experiments L1 stage worms were treated with AuNPs@Citrate, AuNPs@EHL, and EHL, as well as a control group with no treatment. For the treated L1s, the expected dauer larvae and developmental arrest in response to EHL treatment was observed. However, there is no dauer larvae formation in response to AuNPs@Citrate or AuNPs@EHL, and none in the control. As previously reported, EHL also killed L1s in this assay, whilst the other treatments did not differ in L1 survival (see Table 2). From these results, we conclude that AuNPs@Citrate do not obviously affect L1s, and that the AuNPs@EHL do not replicate the EHL effect. In conjunction with the empirical observation that agglutination properties were absent from the AuNPs@EHL sample, this would indicate that a conformational change has occurred potentially in the protein induced by conjugation to the nanoparticles, which blocks the EHL neuronal binding effects.

**Figure 6:**
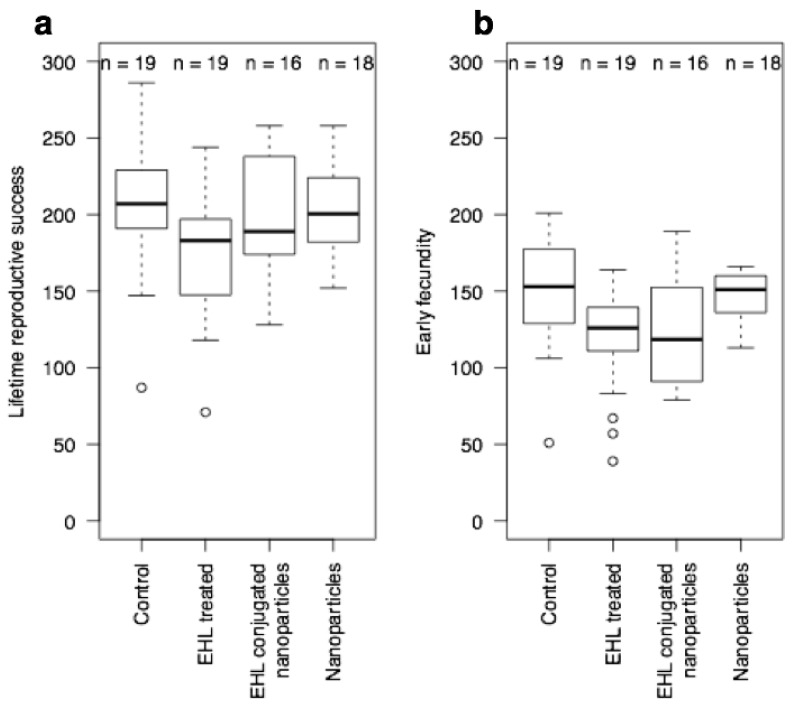
EHL conjugated nanoparticles (AuNPs@EHL) affect early reproduction (**b**), but not total reproduction (**a**) of *C. elegans* L4 stage.

In the second set of experiments, using L4 stage worms, there was a small effect on lifetime fecundity in the EHL treated worms, and a delay in reproduction in both the EHL treated and the AuNPs@EHL worms; whilst naked nanoparticles did not obviously affect the L4 stage worms. We can thus conclude and that AuNPs@EHL does have a biological activity. For the treated L4s, there was a difference between treatments in lifetime fecundity (TREATMENT: F_3,68_ = 2.76, *p* = 0.049) that is a consequence of a small reduction in lifetime fecundity in the EHL treated worms (EHL treatment significantly different to the control and nanoparticle-treated worms by Fisher’s post-hoc testing) (Figure 6, panel A). There is also variation between treatments in early reproduction (H = 14.22, df = 3, *p* = 0.003), with both the EHL treated and the EHL conjugated nanoparticle treated worms showing a reduced early fecundity (*p* < 0.05 in comparison to control worms via Mann-Whitney test) (Figure 6, panel B).

The L4 assays conducted show that AuNPs@EHL do still retain some activity, suggesting that ingestion in the absence of glyconjugate binding (which is absent in the L1 assay) of the molecule may present a low level of toxicity. The observation that AuNPs@EHL do not agglutinate erythrocytes would suggest that this is a factor. Of course, the inverse hypothesis may also be worthy of investigation, that the toxic A-chain activity may have been altered and that binding still occurs without the cytotoxic effects seen in the intact molecule. As non-RIP lectins have been shown to bind to epithelial cells in the gut causing reduced fitness [42], this requires further study to establish the exact reason for the reduction in toxicity.

## 4. Conclusions

The synthesis and characterization of gold nanoparticles conjugated with EHL (AuNPs@EHL) was successfully carried out; optimal functionalization was achieved with the addition of 100 µL of EHL (concentration 1090 ± 40 µg/mL) on 5 mL of AuNPs@Citrate ([Au^0^] = 0.8 mM). Biological assays on the effect of AuNPs@EHL on *C. elegans* were performed, using first life stage (L1) and pre-adult stage (L4) nematodes, and compared to the effect of naked gold nanoparticles and EHL alone. This work shows that the activity of EHL is altered by conjugation and as such resulted in a lessened biological effect towards L1 stage worms. For the assays performed with L4 nematodes, naked nanoparticles do not produce any obvious effect on the worms, while AuNPs@EHL conjugated nanoparticles do produce a similar biological activity to that produced by EHL alone. This indicates that the biological effects of EHL can be separated. Extension of this work to cell lines would therefore be of interest, particularly to determine the mechanism by which the non-toxic effects on L4 worms are produced.

## Figures and Tables

**Table 1:** AuNPs@EHL solution composition for each experiment, DLS, and Zeta Potential Values and protein amount on the nanoparticles ([EHL] = concentration of EHL).

AuNPs@EHL Sample	1	2	3	4	5	6
Vol. EHL added (µL)	25	50	100	200	300	500
Total vol. of the reaction (µL)	5025	5050	5100	5200	5300	5500
Mass EHL in the reaction (µg)	27.3	54.5	109	218	327	545
Mass EHL in supernatant (µg/mL)	3.1 ± 0.1	7.4 ± 0.3	4.0 ± 0.1	40 ± 2	61.1 ± 0.4	84 ± 3
Mass EHL in VT supernatant (µg)	15.6 ± 0.5	37.4 ± 0.3	20.4 ± 0.1	208.0 ± 10	323.8 ± 2	462.0 ± 17
[EHL] in the NPs (µg)	11.7	17.1	88.6	10.0	3.2	83.0
Z-Average value (nm)	266.4	90.3	54.4	51.7	60.8	51.3
Polydispersity Index (PDI)	0.29	0.26	0.28	0.42	0.44	0.44
Zeta Potential (mV/cm)	−23.1	−19.8	−27.8	−24.6	−20.2	−29.4

EHL, *Eranthis hyemalis* lectin.

**Table 2:** EHL treatment affects survival and development of treated *C. elegans* L1s. When scored, adult worms were only observed on 3 of the 12 EHL plates, with worms showing varied degrees of developmental delay.

Treatment	No. of Plates	L1s per Plate	Mean % Survival (min. and max.)	Mean % Dauer Formation (min. and max.)
Control	11	54.6 ± 3.8	68 (53–81)	0
EHL	12	64.2 ± 7.0	23 (11–40) *	24 (0–45) *
AuNPs@Citrate	11	58.6 ± 3.2	73 (54–84)	0
AuNPs@EHL	12	48.7 ± 6.6	68 (49–83)	0

* Denotes treatments where the proportion of surviving worms or dauer larvae differs from that observed in the Control (*p* < 0.05, by Mann-Whitney for the analysis of % survival and by Fisher’s Exact Test with for the analysis of % dauer larvae formation, both with Bonferroni adjustment to correct for multiple testing).

## Supplementary Materials

The following are available online at http://www.mdpi.com/1996-1944/11/8/1363/s1, Figure S1: Spectroscopic profile of AuNPs@Citrate (Dilution Factor = 1:10), Figure S2: Size distribution measured by Dynamic Light Scattering. Distribution by % Intensity and % Volume, Figure S3: Size distribution measured by Dynamic Light Scattering of AuNPs@EHL-3 in water and PBS. Distribution by %Intensity and %Volume.
