# Supplementary material for: Synthesis of Gold Functionalised Nanoparticles with the Eranthis hyemalis Lectin and Preliminary Toxicological Studies on Caenorhabditis elegans
Source: Materials (Basel). 2018 Aug 6;11(8):1363. doi: 10.3390/ma11081363 (PMC6119933; doi:10.3390/ma11081363)
Supplement: Supplementary file 1 [file materials-11-01363-s001.pdf]

materials

MDPI

Article

Synthesis of Gold Functionalised Nanoparticles with the *Eranthis Hyemalis* lectin and toxicological studies on *Caenorhabditis Elegans*.

Jamila Djafari [1,2], Marie T. McConnell [3], Hugo M. Santos[1,2], José Luis Capelo [1,2], Emilia Bertolo [3*] Simon C. Harvey [3], Carlos Lodeiro [1,2], and Javier Fernández-Lodeiro [1,2*]

[1] BIOSCOPE Group. LAQV@REQUIMTE. Chemistry Department. Faculty of Science and Technology. University NOVA of Lisbon. Caparica Campus. 2829-516 Caparica. Portugal.
[2] PROTEOMASS Scientific Society. Rua dos Inventores. Madam Parque. Caparica Campus. 2829-516 Caparica. Portugal.
[3] Biomolecular Research Group. School of Human and Life Sciences. Canterbury Christ Church University. Canterbury. Kent CT1 1QU. UK.
* Correspondence: emilia.bertolo@canterbury.ac.uk and j.lodeiro@fct.unl.pt

1. Gold (0) concentration in colloid precursor.

Concentration of Gold(0) in the colloid precursor was obtained by UV-Vis measurements at = 400 nm [1–3]. For this wavelength, the absorption is mainly due to interband transitions in metallic gold [3, 4]. We applied a 1:10 dilution to the colloidal solution to obtain the UV-Vis spectroscopic profile, obtaining an Abs_{400} = 0.19, corresponding to concentration of [Au(0)] = 0.08 mM. Therefore the precursor colloid used for the protein conjugation presented a concentration of 0.8 mM.

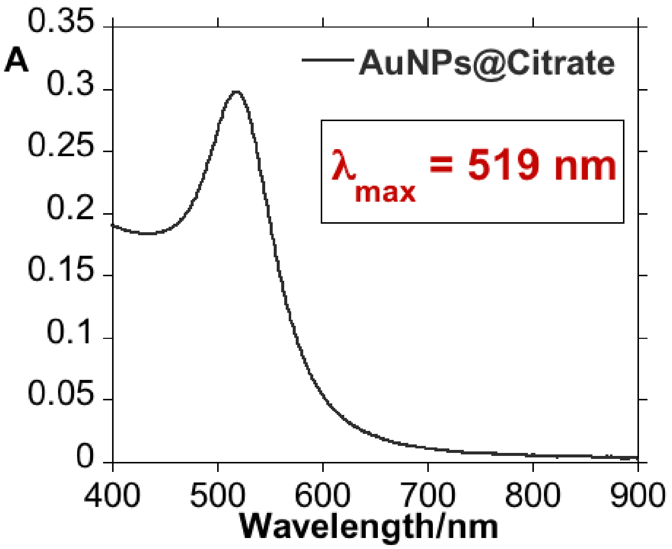

Figure S1: Spectroscopic profile of AuNPs@Citrate (Dilution Factor = 1:10).

2. Dynamic light scattering (DLS) studies.

To try to observe, using the DLS analysis, the possible excess of EHL in the Gold colloids, we first analyzed a suspension of EHL in water (25 L of EHL solution in PBS (C = 1.09 mg / mL) dispersed in 5 mL of ultrapure water). To register the DLS measurement of EHL, we did not filter

this solution and we defined the same parameters used in the colloids analysis (predefined parameters corresponding to Gold colloids) to keep constant the measurement conditions in all samples (EHL free and Gold colloids).

As expected, by dispersing the EHL protein in water, the DLS obtained has two well-differentiated signals. The signal that appears to a smaller diameter is likely to originate from free EHL protein units in solution, while the larger one could be related to EHL aggregates in the aqueous solution.

When comparing the data of the pure EHL protein with the functionalized colloids, it can be noted that the sample AuNPs@EHL-3 presents the best functionalization, not detecting excess protein for this case (figure 2S). Additionally, the analysis of the DLS in water or PBS for this sample offers a similar result in both media (figure 3S).

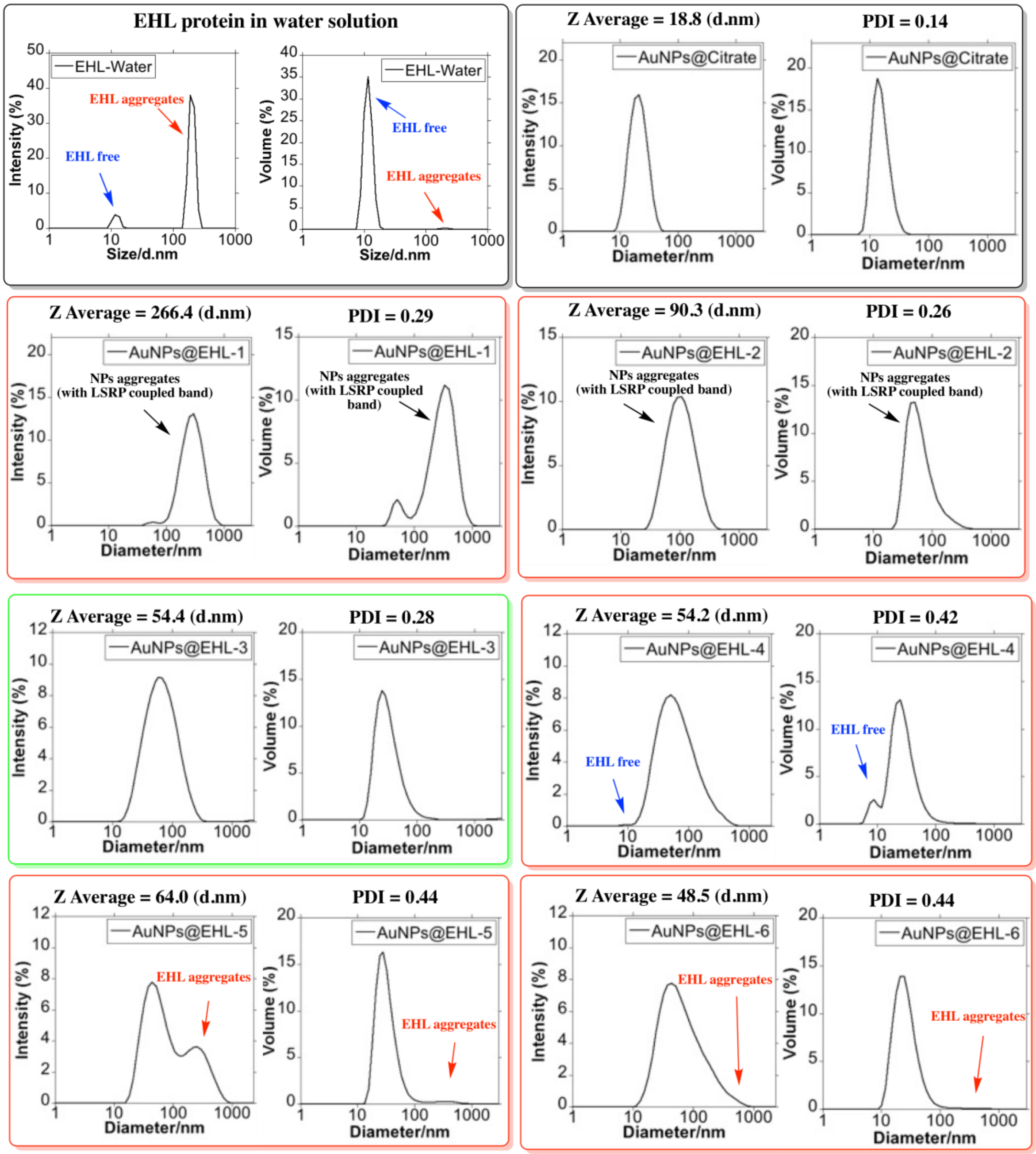

Figure S2: Size distribution measured by Dynamic Light Scattering. Distribution by %Intensity and %Volume.

materials MDPI

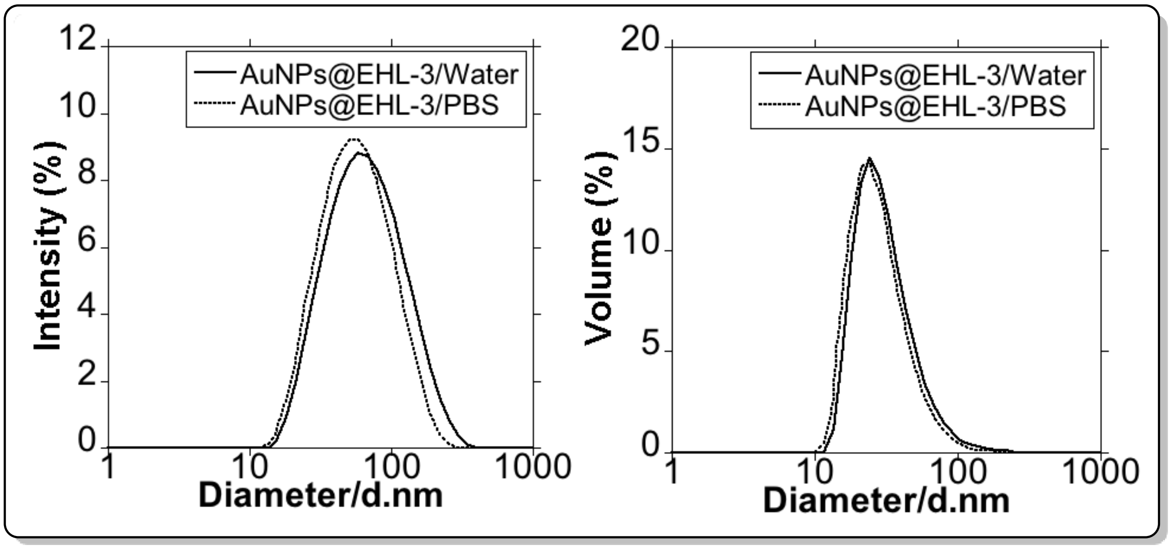

Figure S3: Size distribution measured by Dynamic Light Scattering of AuNPs@EHL-3 in water and PBS. Distribution by %Intensity and %Volume.

Based on our analysis of UV-Vis, DLS and TEM, we feel that the sample AuNPs @ EHL-3 presents a correct functionalization with EHL, without apparent excesses of protein not adsorbed on the surface of the NPs. In addition, during the purification of gold colloids by centrifugation, the remaining NPs present in the first supernatant in samples obtained for larger amounts of protein used (AuNPs-4 to AuNPs-6), making the protein quantification, using colorimetric techniques, can be strongly affected by the presence of this remnant of NPs in the solution, producing data of doubtful quantification. Based on all these considerations, we selected the sample AuNPs@EHL-3 to complete the proposed biological studies, in order not to mask possible results that would be consequence of non-correctly adsorbed protein in the NPs.

3. References

1 Scarabelli, L.; Sánchez-Iglesias, A.; Pérez-Juste, J.; Liz-Marzán, L. M. A 'Tips and Tricks' Practical Guide to the Synthesis of Gold Nanorods. J. Phys. Chem. Lett. 2015, 6 (21), 4270–4279. DOI: 10.1021/acs.jpclett.5b02123

2 Hendel, T.; Wuithschick, M.; Kettemann, F.; Birnbaum, A.; Rademann, K.; Polte, J. In Situ Determination of Colloidal Gold Concentrations with Uv-Vis Spectroscopy: Limitations and Perspectives. Anal. Chem. 2014, 86 (22), 11115–11124. DOI: 10.1021/ac502053s

3 Scarabelli, L.; Grzelczak, M.; Liz-Marzán, L. M. Tuning Gold Nanorod Synthesis through Prereduction with Salicylic Acid. Chem. Mater. 2013, 25 (21), 4232–4238. DOI: 10.1021/cm402177b

4 Rodríguez-Fernández, J.; Pérez-Juste, J.; Mulvaney, P.; Liz-Marzán, L. M. Spatially-Directed Oxidation of Gold Nanoparticles by Au(III)-CTAB Complexes. J. Phys. Chem. B 2005, 109 (30), 14257–14261. DOI: 10.1021/jp052516g
